# 2′-O methylation of RNA cap in SARS-CoV-2 captured by serial crystallography

**DOI:** 10.1073/pnas.2100170118

**Published:** 2021-05-10

**Authors:** Mateusz Wilamowski, Darren A. Sherrell, George Minasov, Youngchang Kim, Ludmilla Shuvalova, Alex Lavens, Ryan Chard, Natalia Maltseva, Robert Jedrzejczak, Monica Rosas-Lemus, Nickolaus Saint, Ian T. Foster, Karolina Michalska, Karla J. F. Satchell, Andrzej Joachimiak

**Affiliations:** ^a^Center for Structural Genomics of Infectious Diseases, Consortium for Advanced Science and Engineering, University of Chicago, Chicago, IL 60637;; ^b^Department of Biochemistry and Molecular Biology, University of Chicago, Chicago, IL 60637;; ^c^Department of General Biochemistry, Faculty of Biochemistry, Biophysics and Biotechnology of Jagiellonian University, Krakow 30387, Poland;; ^d^Structural Biology Center, X-Ray Science Division, Argonne National Laboratory, Lemont, IL 60439;; ^e^Center for Structural Genomics of Infectious Diseases, Feinberg School of Medicine, Northwestern University, Chicago, IL 60611;; ^f^Data Science and Learning Division, Argonne National Laboratory, Lemont, IL 60439

**Keywords:** Nsp10/16, SARS-CoV-2, mRNA, CAP-1, serial crystallography

## Abstract

The genome of the severe acute respiratory syndrome coronavirus 2 (SARS-CoV-2) coronavirus has a capping modification at the 5′-untranslated region (UTR) to prevent its degradation by host nucleases. These modifications are performed by the Nsp10/14 and Nsp10/16 heterodimers using S-adenosylmethionine as the methyl donor. Nsp10/16 heterodimer is responsible for the methylation at the ribose 2′-O position of the first nucleotide. To investigate the conformational changes of the complex during 2′-O methyltransferase activity, we used a fixed-target serial synchrotron crystallography method at room temperature. We determined crystal structures of Nsp10/16 with substrates and products that revealed the states before and after methylation, occurring within the crystals during the experiments. Here we report the crystal structure of Nsp10/16 in complex with Cap-1 analog (^m7^GpppA_m2′-O_). Inhibition of Nsp16 activity may reduce viral proliferation, making this protein an attractive drug target.

## Significance

The 2′-O methyl group in Cap-1 is essential to protect viral RNA from host interferon-induced response. We determined crystal structures of SARS-CoV-2 Nsp10/16 heterodimer in complex with substrates (Cap-0 analog and S-adenosyl methionine) and products (Cap-1 analog and S-adenosyl-L-homocysteine) at room temperature using synchrotron serial crystallography. Analysis of these structures will aid structure-based drug design against 2′-O-methyltransferase from SARS-CoV-2.

There are three main pathogenic coronaviruses that cause major respiratory diseases in humans: severe acute respiratory syndrome coronavirus (SARS-CoV), Middle East respiratory syndrome coronavirus (MERS-CoV), and severe acute respiratory syndrome coronavirus 2 (SARS-CoV-2) (1, 2). SARS-CoV-2 (the cause of COVID-19) has spread rapidly around the world, infecting, at the time of manuscript preparation, over 128 million people and killing over 2.8 million, all while the number of cases are continuing to increase (3). Despite the roll out of several SARS-CoV-2 vaccines, finding inhibitors of a biological cycle of the virus is still critical, especially due to mutations that could be resistant to vaccines.

SARS-CoV-2 β-coronavirus has a large (∼30 kb) and complex (29 proteins) (+) sense single-stranded RNA genome (4). The RNA in the mature virion resembles human mRNA: 1) it is capped on its 5′-end, 2) it contains a 3′-poly-A tail, and 3) after infection, it can be directly translated to the two polyproteins Pp1a and Pp1ab using host machinery. These polyproteins then mature and are cut into 16 polypeptides and 15 nonstructural proteins (Nsps) that assemble into a large replication–transcription complex. The RNA is also used as a template for the biosynthesis of (−) sense RNA that serves to make additional copies of (+) sense RNA and several subgenomic RNAs to translate structural and accessory proteins (4). These RNAs must undergo posttranscriptional modifications to resemble eukaryotic mRNA and protect the transcripts from degradation and improve translation in the human host (5). The RNA capping involves several enzymatic steps performed by viral Nsps; Nsp13 is a bifunctional RNA/NTP triphosphatase and helicase that hydrolyses the first phosphate from the nascent RNA; then an unknown guanylyltransferase transfers a guanosine monophosphate moiety to the pp-RNA forming Gppp-RNA. This is followed by transfer of a methyl group from the donor S-adenosylmethionine (AdoMet) to the guanidine N7 to form ^m7^GpppA_2′-OH_-RNA (Cap-0-RNA) by Nsp10/14 heterodimer. Finally, a metal-dependent Nsp10/16 heterodimer methylates the ribose 2′-O of the first nucleotide (usually adenosine, in coronaviruses) of the nascent mRNA Cap-0-RNA to form Cap-1-RNA (^m7^GpppA_m2′-O_-RNA) using AdoMet as the donor (6). The 2′-O methylation of the Cap-0-RNA prevents activation of type I interferon-induced response by the cytoplasmic RNA sensors: melanoma differentiation-associated protein 5 (MDA5), and retinoic acid-inducible gene-I (RIG-1) (7–9). Mutations in 2′-O methyltransferase render the viruses sensitive to the interferon-inducible immunological pathways (10). Therefore, inhibition of the Nsp16 activity may reduce viral proliferation, making the protein an attractive drug target.

Methyltransferases are common in all metazoan and viral genomes (11). The sequence identity of Nsp16 between SARS-CoV-2 and SARS-CoV-1 is 95%, while the identity of SARS-CoV-2 Nsp16 to the MERS-CoV enzyme is 66% (12). The active site of Nsp16 MTase is conserved in the Coronaviridae family; they all utilize a Lys-Asp-Lys-Glu catalytic tetrad essential for the enzymatic activity (13, 14).

While several structures of Nsp10/16 in a complex with Cap-0 analog have been determined by single crystal cryocrystallography (Protein Data Bank [PDB] entries: 6WQ3, 6WRZ, 6WVN, 6WKS, 6YZ1, 6WKQ, 6WJT, 6W61, 6W75, 6W4H, 7BQ7, 7C2I, and 7C2J) (12, 15–17), none has been determined at room temperature. We conducted serial synchrotron crystallography (SSX) (18) experiments to capture the 2′-O methyl transfer reaction, using the Cap-0 analog (^m7^GpppA) and AdoMet as substrates. The SSX is a synchrotron equivalent of an X-ray free-electron laser serial femtosecond crystallography experiment and uses a similar sample delivery approach. Here, we present three crystal structures of the Nsp10/16 complex determined by fixed-target SSX at 295 K in the presence of AdoMet, Cap-0/AdoMet, and with Cap-1/S-adenosyl-L-homocysteine (AdoHcys). The metal-dependent 2′-O methyl transfer catalysis was observed in the crystals. We compare these structures with structures of Nsp10/16 at 295 K and 100 K collected from a single crystal.

## Results and Discussion

### Serial Synchrotron Crystallography of the Nsp10/16 2′-O MTase from SARS-CoV-2.

**Fig. 1.**
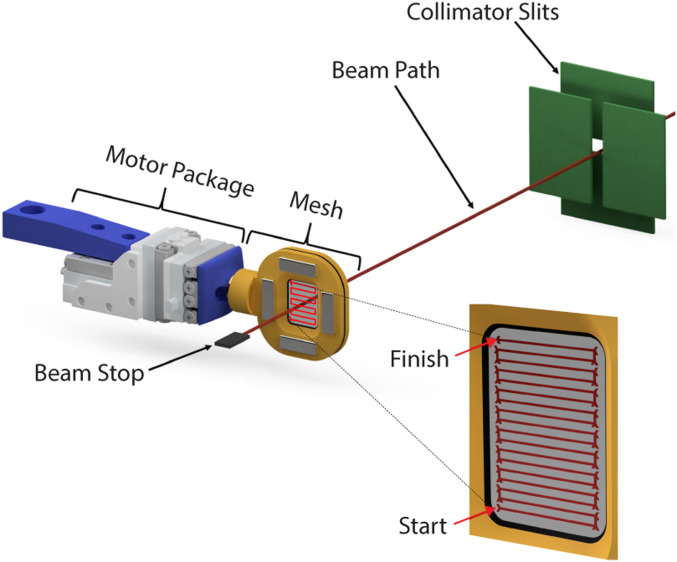
Fixed-target serial crystallography data collection on Nsp10/16 crystals from SARS-CoV-2. System for SSX with moving crystal holder developed at SBC beamline 19ID.

We collected SSX data for the Nsp10/16 crystals using the fixed-target SSX system (Fig. 1 and *SI Appendix*, Figs. S1 and S2). A crystal slurry is deposited on a nylon mesh and sandwiched between two polyester films (referred here as a chip), which immobilizes the crystals (19). The chip is then rastered through the X-ray beam. Representative diffraction images recorded from single Nsp10/16 crystals illustrate the obtained data (*SI Appendix*, Figs. S1 and S2). We collected diffraction data from seven chips with ∼36,100 images per chip with an exposure time of 50 ms at 100% X-ray transmission (*SI Appendix*, Fig. S2). We used the Kanzus automated pipeline for data processing. Two structures were solved at 2.25 Å (Nsp10/16/AdoMet and Nsp10/16/Cap-1/AdoHcys), and one at 2.18 Å (Nsp10/16/Cap-0/AdoMet) (*SI Appendix*, Table S1). Structure of Nsp10/16/Cap-1/AdoHcys was obtained when crystals of Nsp10/16/Cap-0/AdoMet were incubated with solution containing Mn^2+^ ions. The highest resolution shell in PRIME analysis was specified using a CC_1/2_ cutoff below 0.40. Data completeness was nearly 100% (*SI Appendix*, Table S2), and after the structure refinements, R_work_ was 20.65%, 22.31%, and 21.70% for Nsp10/16/AdoMet, Nsp10/16/Cap-1/AdoHcys, and Nsp10/16/Cap-0/AdoMet, respectively (*SI Appendix*, Table S2). For a comparison with SSX data, we collected diffraction data from a single crystal of the Nsp10/16 containing both Cap-0/AdoMet and Cap-1/AdoHcys in a capillary at 295 K. These data were processed to 2.65 Å (*SI Appendix*, Tables S1 and S2). Using RADDOSE-3D (20), we estimated the accumulated dose of X-rays for Nsp10/16 from SSX experiments to be 0.12 MGy/dataset, whereas the dose for the capillary-mounted crystal was 4.97 MGy: over 40 times higher. And this could be a reason why the diffraction resolution for the last one dataset was lower.

### Structure of the Nsp10/16 Heterodimer from SARS-CoV-2 at 295 K.

**Fig. 2.**
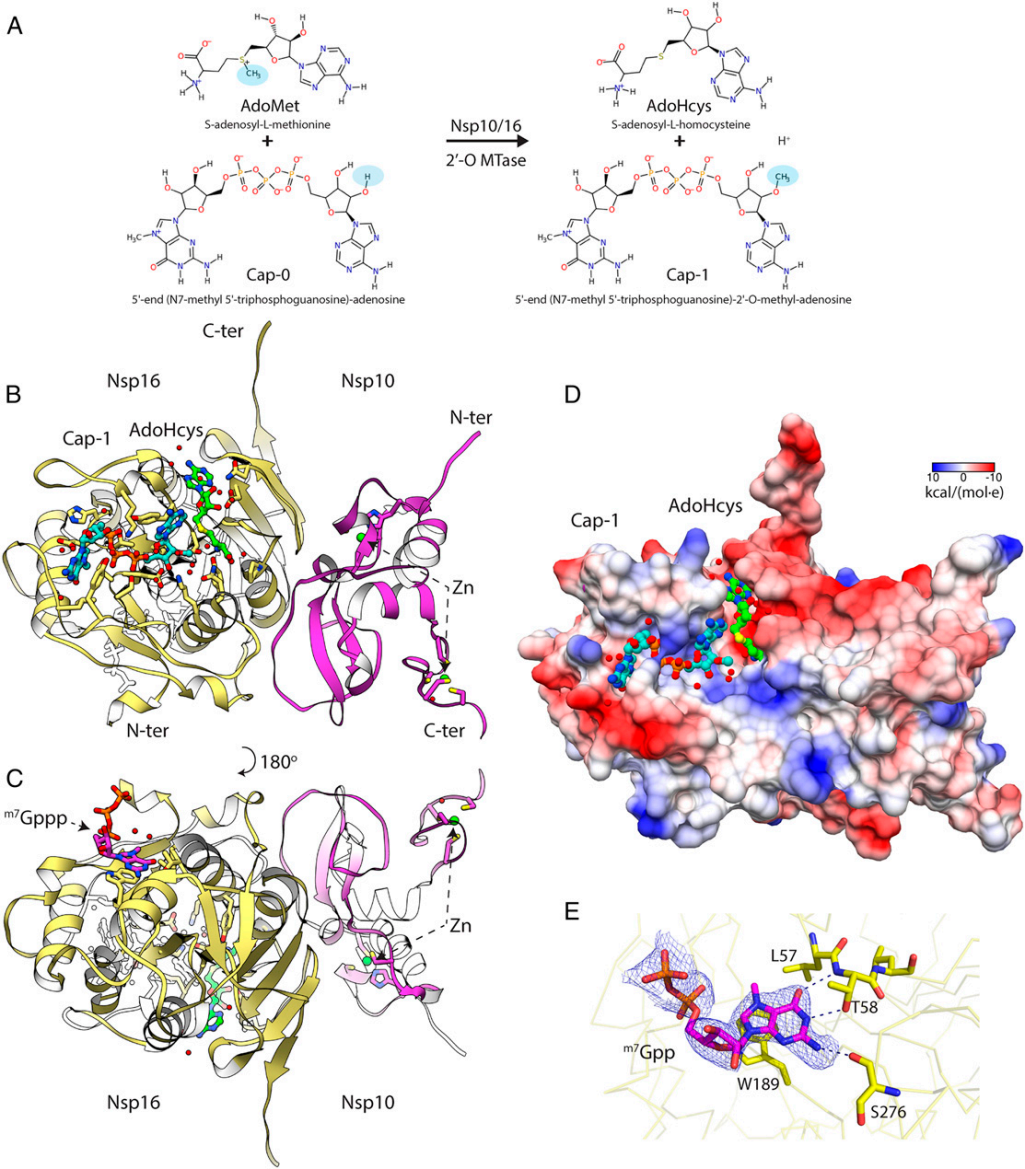
Crystal structure of the Nsp10/16 in a complex with ^m7^GpppA_m2′-O_ (Cap-1) and AdoHcys determined using fixed-target SSX at 295 K. (*A*) Diagram of the 2′-O methyl transfer reaction performed by Nsp10/16; methyl groups are highlighted with blue ovals. (*B*) The assembly of the Nsp10/16 heterodimer; Nsp10 (pink), Nsp16 (yellow) in complex with Cap-1 (aquamarine), and AdoHcys (green) (PDB entry 7JHE). (*C*) The ^m7^Gppp at the potential allosteric site of Nsp16. (*D*) The Coulombic electrostatic potential mapped on the surface of the Nsp10/16 heterodimer in complex with Cap-1/AdoHcys at 295 K calculated using default settings in UCSF Chimera. (*E*) 2mFo-DFc composite omit map calculated for ^m7^Gpp and contoured at 1.2 σ (PDB entry 7JPE). The Nsp16 is shown as yellow ribbon, selected residues (yellow), and ^m7^Gpp are depicted as pink sticks.

The Nsp10/16 complex is an α/β heterodimer (Fig. 2). The 139-amino-acid-long Nsp10 has a unique fold formed by 5 α-helices and a pair of antiparallel β-strands which are facing Nsp16 in the complex. Nsp10 possesses two zinc ions coordinated by the Cys74, Cys77, His83, Cys90 and Cys117, Cys120, Cys128, Cys130 motifs that are 100% conserved in β-coronaviruses (12). Nsp10 is involved in forming complexes with Nsp14 3′–5′ exoribonuclease/guanine N7 methyltransferase and Nsp16 2′-O-ribose MTase. Previous research showed that Nsp10 is required for Nsp16 activity (21). Nsp10 is a cofactor for two SARS-CoV-2 enzymes and therefore a good candidate for design of molecules that could affect its structure or disrupt the interaction with Nsp14 or Nsp16 2′-O MTases.

The structure of Nsp16 has a Rossmann-like fold, with a large β-sheet surrounded by α-helices, β-strands, and loops. Nsp16 contains 11 α-helices, 12 β-strands, and 7 loops. Nsp16 has a centrally positioned β-sheet (β3↑,β2↑,β1↑,β4↑,β5↑,β7↓,β6↑) with only one antiparallel strand β7. The AdoMet and the Cap-binding sites are located at the surface of Nsp16. None of the Nsp10 residues have a direct connection with the binding sites of the ligands, although SAM binding seems to recruit Nsp10 in MERS (22) (Fig. 2 *B* and *C*).

Analysis of the surface electrostatic charges of the Nsp10/16 shows positively charged residues that form an elongated binding pocket for the Cap (the high-affinity binding site), with approximate length of 21 Å (Fig. 2*D*). In proximity of the Cap-0 methylation site there is another elongated positively charged surface that can potentially bind longer RNA (low-affinity binding site) (Fig. 2*D*). Recently deposited structures of Nsp10/16 with (^m7^GpppA)pUpUpApApA (PDB entries 7JYY, 7JZ0, 7L6R, and 7L6T) show positions of three more nucleotides, UUA, located in this positively charged region of Nsp10/16. The remaining two adenosine bases at the 3′-terminus of the RNA are disordered. Moreover, there are reports that two zinc fingers of Nsp10 contribute to nonspecific binding of nucleic acids (6), which could further indicate that Nsp10/16 not only binds the Cap, but also interacts with longer RNA fragments. We observed the structure of Nsp10/16 with substrates and products of the reaction as well as electron density for the partially occupied 7-methyl-guanosine-5′-diphosphate (^m7^Gpp) of the Cap in the possible allosteric site of Nsp10/16 (Fig. 2*E*) as previously described (12, 15).

**Fig. 3.**
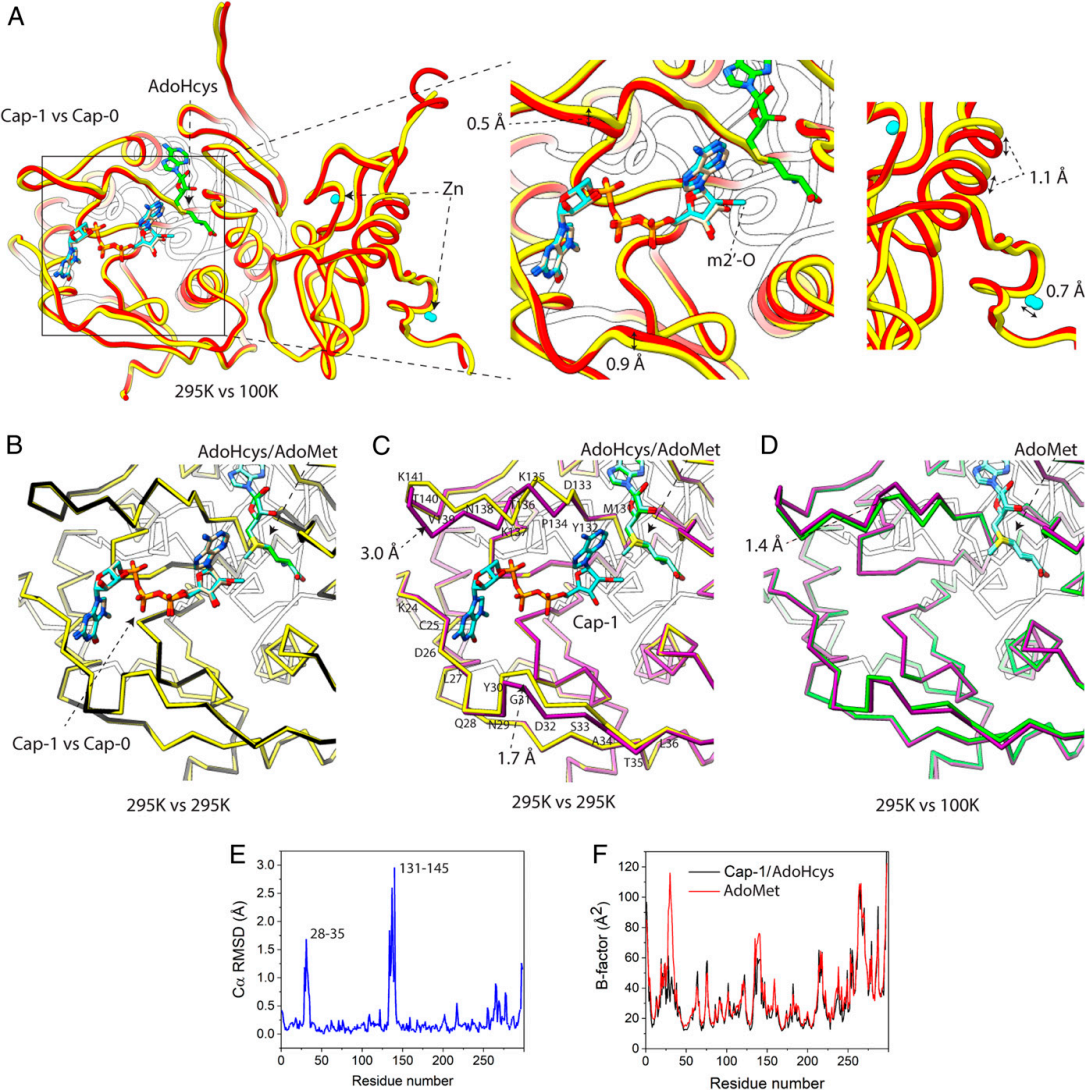
Comparison of crystal structures of the Nsp10/16 determined at 295 K and at 100 K. (*A*) Crystal structure of the Nsp10/16 with Cap-1 and AdoHcys (yellow, 7JHE) determined using SSX superimposed with Nsp10/16 with Cap-0 and AdoHcys (red, 6WQ3) determined at 100 K. (*B*) Comparison of 295 K crystal structures of Nsp10/16/Cap-1/AdoHcys (yellow, 7JHE) superimposed to the structure of Nsp10/16/Cap-0/AdoMet (black, 7JPE). (*C*) Comparison of 295 K crystal structures of the Nsp10/16/Cap-1/AdoHcys (yellow, 7JHE) superimposed to the structure of Nsp10/16/AdoMet (pink, 6XKM). (*D*) Comparison of the Nsp10/16 crystal structures with AdoMet determined respectively using SSX (pink, 6XKM) and standard data collection at 100 K (green, 6W4H). (*E* and *F*) Binding of Cap-1 to Nsp16. Plots of rmsd (*E*) and B factor of structures with AdoMet and Cap-1/AdoHcys (*F*).

**Fig. 4.**
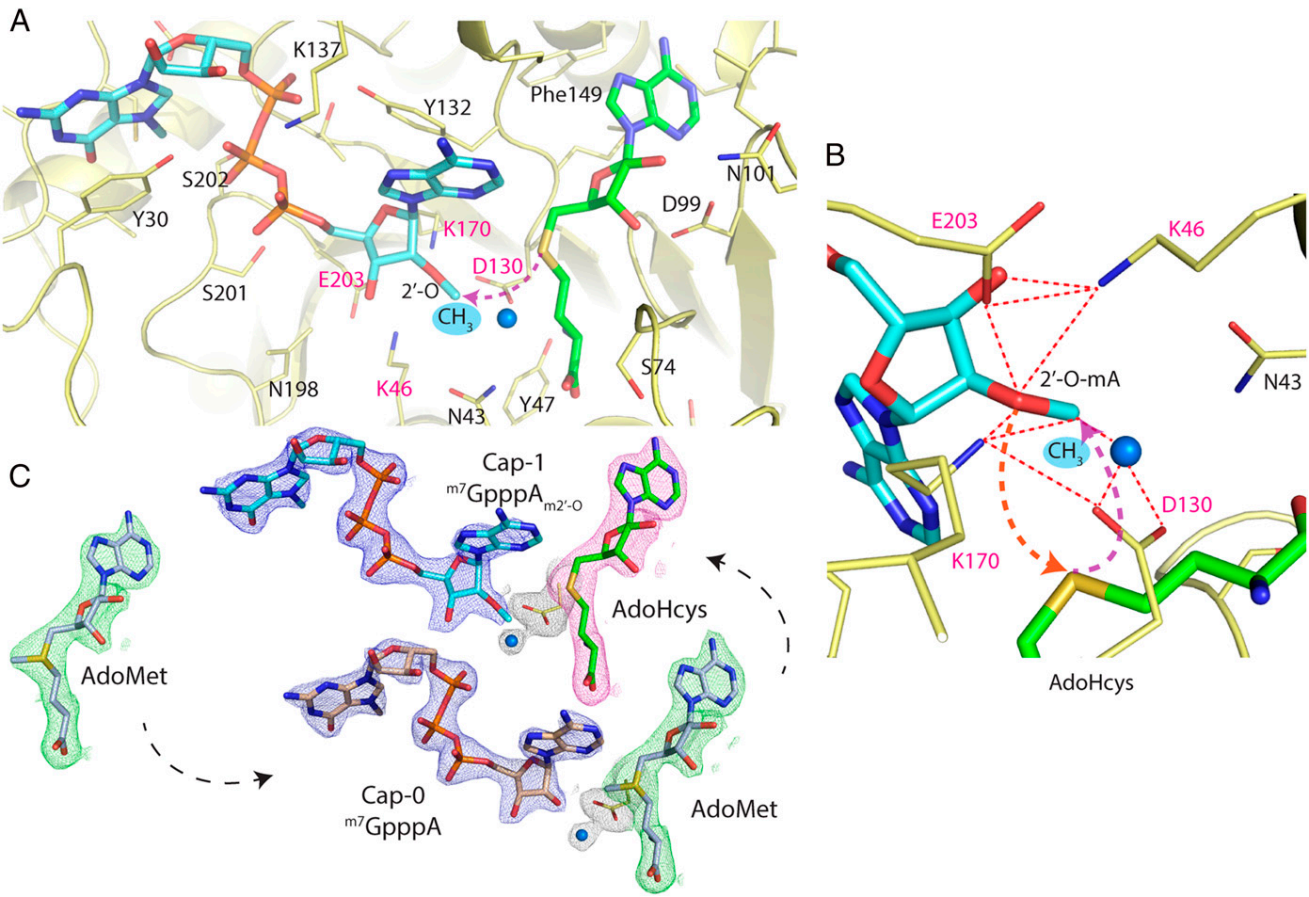
Methylation of the 2′-O-ribose of the ^m7^GpppA catalyzed by Nsp10/16 from SARS-CoV-2. (*A*) Active site of Nsp16 2′-O methyltransferase (yellow) in complex with ^m7^GpppA_m2′-O_ (Cap-1) as aquamarine sticks and AdoHcys as green sticks. (*B*) Magnification of the active site depicts catalytic tetrad Lys46-Asp130-Lys170-Glu203 essential for the 2′-O MTase activity shown as yellow sticks. Red dashed lines show close distances between residues and arrows depict simplified nucleophilic attack and subsequent movement of methyl group to the 2′-O position. The water molecule is represented as a blue sphere. (*C*) The 2mFo-DFc maps contoured at 1.2 σ around ligands of the structures determined by SSX; cap analogs are in blue, AdoHcys in pink, and AdoMet in green.

The SSX experiments using the Nsp10/16 crystals revealed three states: one with AdoMet bound, the second with AdoMet and Cap-0 in the active site prior to the methyl transfer reaction, and the third with Cap-1 and AdoHcys after methyl transfer (Fig. 3 and *SI Appendix*, Fig. S3). The electron density clearly showed the methyl group present on the adenosine moiety at the 2′-O-ribose position of the Cap-1 and no methyl present on AdoHcys (Fig. 4*C* and *SI Appendix*, Fig. S3). Moreover, the single crystal structure determined at 295 K has also clear electron density for the methyl moiety on the Cap 2′-O-ribose, but with partial occupancy. This structure represents a mixture of two states, before (70%) and after (30%) reaction.

The structures determined at 100 K using a single crystal and at 295 K using SSX are very similar, as illustrated by superpositions of our Nsp10/16/Cap-1/AdoHcys structure with the complex of Cap-0/AdoHcys (PDB entry 6WQ3) (Fig. 3*A*). For these structures, the overall rmsd of Cα atoms is 0.43 Å. We observed less than a 1.10-Å shift of the loops that form the binding sites for the Cap. Comparison of the SSX structures of Nsp10/16 with Cap-0/AdoMet and Cap-1/AdoHcys does not reveal any significant changes in protein backbone (Cα rmsd: 0.52 Å) (Fig. 3*B*). On the other hand, the comparison of Nsp10/16/AdoMet and Nsp10/16/Cap-1/AdoHcys complexes determined using SSX indicate larger conformational changes in the Nsp16 that are induced by the Cap-0 binding and Cap-1 formation. We observed movement of the two loops (comprising residues 28 to 35 and 131 to 146 of Nsp16), which form the capped mRNA-binding groove; positions of some residues are shifted as much as 3.00 Å, with an overall Cα rmsd of 0.40 Å (Fig. 3 *C*, *E*, and *F*). At the same time, the Cap binding did not significantly effect the structure of Nsp10. We also compared the two structures of Nsp10/16/AdoMet determined at 295 K (this work PDB entry 6XKM) and at 100 K (PDB entry 6W4H) (Cα rmsd: 0.35 Å) and observed significant differences: the segment of the loop (residues 138 to 146) and the short α-helix (residues 134 to 137) forming a part of the Cap-binding pocket are shifted ∼1.40 Å (at Lys135) in structures at 295 K, which makes the Cap-binding site more accessible (Fig. 3*D*). There are other Nsp10/16 structures reporting even larger Lys135 movement (15). Therefore, the structures of Nsp10/16 in complex with AdoMet, Cap-0/AdoMet, and Cap-1/AdoHcys, as determined by SSX at 295 K, depict states important for molecular modeling studies and structure-based drug design.

The Mg^2+^ or Mn^2+^ ion is necessary for Nsp16 activity (22). None of our structures indicate metal binding. NS5 2′-O MTase from dengue virus has Mg^2+^ ion bound to the N-terminal domain. We compared our structures with NS5 2′-O MTase from dengue virus (PDB entry 5DTO) (*SI Appendix*, Fig. S4). Despite low sequence identity (13.6%) the rmsd between the two MTases is reasonable (3.01 Å). Both enzymes possess a canonical 2′-O MTase catalytic tetrad Lys-Asp-Lys-Glu with the aspartic acid residue forming a hydrogen bond with a water molecule potentially relevant for catalysis (*SI Appendix*, Fig. S4 *A* and *C*). The AdoMet-binding sites share high similarity but there are major differences in the mRNA Cap-binding sites. The Mg^2+^ ion in NS5 MTase is coordinated by the phosphate oxygens of the Cap 5′-to-5′ triphosphate linker and three water molecules that bridge to three bases (A_1_-G2-U_3_) on the 5′-end. It is possible that a similar site exists in the Nsp16 enzyme/RNA substrate complex as shown in the structures with Cap-RNA (23).

### 2′-O Methylation of the SARS-CoV-2 Transcripts.

The SARS-CoV-2 Nsp10/16 2′-O MTase complex provides a molecular arrangement for binding of the mRNA Cap-0 and subsequent methylation of the first transcribed nucleotide (Fig. 4). The catalytic core is located in the center of the Rossmann fold and it binds the AdoMet molecule in the deep, narrow groove that is buried inside the Nsp16 active site. We observed that Nsp16 recruited AdoMet during expression in *Escherichia coli* (PDB entry 6W61) (24). The AdoMet-binding site is negatively charged and is formed by several Nsp16 residues: Asn43, Tyr47, Gly71, Ala72, Gly73, Ser74, Gly81, Thr82, Asp99, Leu100, Asn101, Asp114, Cys115, Asp130, Met131, and Phe149 (Figs. 2 and 4 and *SI Appendix*, Fig. S5*A*). The AdoMet carboxylate moiety binds to the positively charged N terminus of the α-helix spanning residues Pro80-Trp88, providing additional electrostatic interaction. The Cap-0 directly binds to Nsp16 through several residues that form a positively charged, elongated binding groove accommodating the mRNA (Figs. 2 and 4 and *SI Appendix*, Fig. S5*B*).

The Nsp16 prefers ^m7^GpppA over GpppA and our structures provide an explanation for this selectivity. The N7-metyl guanosine-binding pocket is formed by Cys25, Asp26, Leu27, Tyr30, Thr172, Glu173, and Ser202. The methylation of guanine at N7 has significant consequences on the property of the base and it allows proper positioning of the Cap-0 in the binding site (Fig. 4*A* and *SI Appendix*, Fig. S3) (15). This is a specific entropic change that introduces a hydrophobic and partially positively charged moiety. Sometimes it is described as cationic guanine and provides new opportunities for interactions. The N7 methyl moiety is well accommodated in the hydrophobic pocket formed by side chain atoms of Leu27 and Cβ carbons of Cys25, Ser202, and Glu173. The side chain oxygen of Glu173 directly, and via a conserved water molecule, interacts with N7 and guanine and neutralizes the positive charge introduced by methylation. Interestingly, our structures revealed several interactions of the base with protein that should be less sensitive to N7 methylation. These include π-stacking with Tyr30 and a H-bond with carbonyl of Cys25. What is remarkable is that all guanine-binding residues are involved in a network of interactions between the protein and the nucleotide, strongly contributing to positioning the Cap-0 (^7m^GpppA) in the active site. Moreover, these residues are conserved in Nsp16s from coronaviruses, including their conformations in Nsp16 structures with AdoMet, Cap-0, and Cap-1 (15, 23). The 5′-to-5′ triphosphate bridge of the Cap is stabilized through interactions with Tyr30, Lys137, Thr172, His174, Ser201, Ser202, and Glu203. The first nucleotide of the mRNA Cap (adenosine in the presented structure) is bound through Lys46, Asp130, Tyr132, Pro134, Lys170, Asn198, and Glu203 (Fig. 4*A*).

AdoMet-dependent MTases share a conserved catalytic mechanism wherein the methyl group is transferred to the acceptor substrate via an S_N_2 reaction (25), which requires a linear alignment of the acceptor substrate (nucleophile), methyl group (electrophile), and the sulfur atom of the AdoHcys product (26). It was proposed that Nsp16 2′-O MTases follow the same general mechanism (27). In these enzymes, the reaction is facilitated by the catalytic tetrad Lys-Asp-Lys-Glu, where Lys170 is sandwiched between the two acidic residues (Asp130 and Glu203) and serves as a proton abstractor (Fig. 4 *A* and *B*). It was shown previously in biochemical studies that substitution with Ala of any residue of the 2′O-MTase catalytic tetrad results in an inactive enzyme (26, 27). In SARS-CoV-2 Nsp16, the catalytic tetrad Lys46-Asp130-Lys170-Glu203 is superposing well with Lys61-D146-Lys180-Glu216 of the dengue virus homolog (*SI Appendix*, Fig. S4 *A* and *C*). Lys170 is well positioned to act as a general base deprotonating 2′-OH. Asp130 may serve multiple functions; as an acid deprotonating Lys170 (alternating with Glu203), an anchoring point for the cofactor via interaction with its amino group, and as a stabilization for the sulfonium cation. However, the reaction does not occur in the presence of ethylenediaminetetraacetic acid (EDTA), as demonstrated by the ability to capture the Cap-0/AdoMet complex (Fig. 4*C* and *SI Appendix*, Fig. S3*A*). It was previously reported using biochemical assays that the activity of Nsp10/16 2′-O MTase is magnesium dependent (22, 28, 29). Despite the presence of magnesium in the crystallization buffer, we did not observe any metal ions near the catalytic site in our structures at 295 K, or in any other SARS-CoV-2 Nsp10/16 structures reported to date. We hypothesize that the magnesium ion (or other metal ion) can transiently bind to the active site or RNA substrate and promote formation of an active conformation by changing electrostatics and geometry of the catalytic residues. Magnesium has a compact and tight coordination sphere with strict octahedral geometry and a typically short Mg–O distance of 2.08 Å (30). By coordinating 2′ oxygen and several water molecules, magnesium could shorten the distance between 2′ oxygen and AdoMet methyl moiety thus promoting formation of a transition state and methyl transfer. During the reaction, a positively charged, sp^2^ planar transition state is formed and the methyl group inverts its stereochemistry.

After the methyl-transfer reaction is completed, the product is released from the active site. This is consistent with the structure containing Cap-1/AdoHcys where several active site residues move, including Tyr132, the entire α-helix spanning Pro134-Lys137, and Tyr30 on the opposite site (Fig. 4*A* and *SI Appendix*, Fig. S3*C*). All these residues are involved in interactions with Cap-0 and observed changes perhaps allow Cap-1 to leave. Interestingly, the AdoMet/AdoHcys-binding site remains virtually unchanged in all three structures (*SI Appendix*, Fig. S3), suggesting that the AdoHcys exchange with AdoMet may require dissociation of Nsp10, which controls conformation of the important Nsp16 loop Gly73-Gly77. Opening of this loop may help AdoHcys to leave and then allow a new AdoMet molecule to bind.

### Conclusions.

Enzymes that catalyze methyl transfer reactions using AdoMet as the methyl group donor have been described in many cellular processes involving nucleic acids, proteins, phospholipids, and small molecules (31). SARS-CoV-2 Nsp16 methyltransferase is one representative of this large family of proteins, which, together with Nsp10, participates in the posttranscriptional modification of the viral (+) RNA from CoV. This multistep RNA maturation process is a prerequisite to yield fully functional mRNA that would not be degraded (32) and can undergo translation in the human host (5, 33). 2′-O methylation of the mRNA Cap facilitated by the Nsp10/16 is the last reaction in mRNA maturation. Given the indispensable role of the RNA modification for the survival of the virus, the disturbance of this pathway, including the 2′-O methylation of Cap, is a field for development of inhibitors that affect SARS-CoV-2 replication. To that end, understanding the structure–function relationship of the enzymes catalyzing these processes is critical.

Here we report four structures of the SARS-CoV-2 Nsp10/16 in complex with cap analogs revealing the prior and post 2′-O methyl transfer reaction states. The SSX experiments with the Nsp10/16 crystals revealed three states: with AdoMet bound, with AdoMet and Cap-0 prior to the reaction, and, finally with Cap-1 and AdoHcys after methyl transfer. We also describe the preference of the enzyme for ^m7^GpppA over GpppA. These experiments done at 295 K under low X-ray radiation dose show the structure of Nsp10/16 with Cap-1, which is a difficult event to capture due to low catalytic activity of Nsp10/16 when the Cap analog is used as a substrate (23). The uniqueness of the Cap-1 structure also shows the advantages of the SSX method in structural studies as it considerably improved resolution, uses less sample in comparison with a liquid jet delivery system, and reduces levels of X-ray dose. These technical advantages could allow the use of larger crystals, reducing radiation damage, and being closer to physiological temperatures. Studies of other enzymes can also significantly benefit from using this approach.

## Materials and Methods

### Serial Crystallography Data Collection.

We collected SSX data for the Nsp10/16 crystals at the 19ID beamline at the Advanced Photon Source using the fixed-target SSX Advanced Lightweight Encapsulation for Crystallography (ALEX) mesh holder developed at the Structural Biology Center (SBC) as depicted in Fig. 1. The diffraction images from all crystals were recorded at 295 K on the PILATUS3 × 6 M detector using still and 0.05-s exposure. A crystal slurry was deposited on a nylon mesh to immobilize the crystals; they were then encapsulated between two polyester films (19). The rod-shape crystals grew to the average size 120 × 25 × 25 µm (*SI Appendix*, Fig. S1*A*). Serial data were collected using three SmarAct SLC-17 stages configured in an XYZ geometry, with each having sufficient movement range to cover the sample area of the specially designed ALEX holder (patent application serial #16/903,601). The beamline was configured at an energy of 12,662 eV, with collimator slit sizes set to 75 × 75 µm, and step size (distance between exposures) of 50 µm, overlapping the exposed area to maximize crystal hits. The five mesh-covered samples used a grid of 170 steps in the x direction (columns) by 210 steps in the y direction (rows), covering a total area of ∼8.5 × 10.5 mm. The number of steps and the resulting area varied slightly per sample, depending on the chip mount position or possible false starts. *SI Appendix*, Table S1 contains details of the number of chips and detector distances used for data collection for Nsp10/16 SSX structures.

Crispy, the data acquisition graphical user interface (GUI) for serial data collection at sector 19, allows for quick alignment and acts as a source of information for downstream processing. Metadata in the form of JSON files and beamline/collection strategy parameters are input and passed into the system before collection. These parameters include grid dimensions, detector distance/resolution, unit cell dimension, protein PDB coordinates, and a handful of others.

### Serial Crystallography Data Processing.

The Kanzus pipeline orchestrates SSX data acquisition, analysis, and cataloging and publishes processing metrics. Kanzus uses a cloud-hosted research automation system called Globus Automate to manage these multistep data “flows” (34). The first phase of the pipeline is integrated with the Advanced Photon Source (APS) Data Management System at the beamline, which deposits each newly acquired image into a Globus-accessible storage system at the APS. As new images are acquired, Globus Automate flows are launched to process them as follows: 1) moves new files from APS to Theta by using the Globus Transfer service (35); 2) performs DIALS *stills_process* (36) on batches of 256 images by using funcX (37), a function-as-a-service computation system [funcX uses Parsl (38) to abstract and acquire nodes on Theta as needed, and dispatches tasks to available nodes]; 3) extracts metadata from files regarding identified diffractions and generates visualizations (funcX) showing the locations of positive hits on the mesh; and 4) publishes raw data, metadata, and visualizations to a portal on the Argonne Leadership Computing Facility (ALCF) Petrel data system (39). The result of this automated process is an indexed, searchable data collection that provides full traceability from data acquisition to processed data that can be used to inspect and update the running experiment.

Images were collected at 7 Hz, meaning that a 256-image batch totaling 1.56 GB was generated every 35 s. Theta is a supercomputer at ALCF 11.69-petaflop system based on the second-generation Intel Xeon Phi “Knights Landing” (KNL) processor. Its 4,392 nodes each have a 64-core processor with 16 GB MCDRAM, 192 GB of DDR4 RAM, and are interconnected with high-speed InfiniBand. The data transfers to ALCF ran at up to 700 MB/s via Globus, and 30 Theta nodes processed images by using DIALS *stills_process* at 22 images per second in steady state. As experimental configuration values were refined, reprocessing tasks were submitted as required. FuncX managed these tasks by expanding the number of Theta nodes being used to a maximum of 250, which enabled a processing rate of greater than 200 images a second. Successfully processed images with diffraction-produced integration files were returned to the beamline computers and later refined and merged using PRIME (40).

### Structure Solution and Refinement.

The crystal structures of the Nsp10/16 complex were solved by molecular replacement using MolRep (41) from the CCP4 package. The structures were refined by multiple cycles in REFMAC v. 5.8.0258 (42) followed by manual corrections of the model using Coot (43). The ligands Cap-1, Cap-0, ^m7^Gppp, ^m7^Gpp, AdoMet, AdoHcys, and Zn^2+^ were manually placed into electron density in Coot and waters were generated using ARP/wARP (44). The stereochemistry of structures was analyzed using MolProbity (45) and the Ramachandran plot. The atomic coordinates and structure factors for Nsp10/16 structures determined with SSX were deposited to PDB with assigned accession codes: 6XKM for AdoMet, 7JPE for Cap-0/AdoMet, and 7JHE for Cap-1/AdoHcys. Nsp10/16 complex with mixture of Cap-0/Cap-1 and AdoHcys/AdoMet determined from a single crystal at 295 K has PDB entry 7JIB. Figures were prepared with PyMol and Chimera (46).

## Supplementary Material

Supplementary File

## Data Availability

Structure data have been deposited in the Protein Data Bank (6XKM, 7JHE, 7JPE, and 7JIB) (47–50).

## Change History

September 1, 2021: The article text has been updated.
