# Supplementary material for: 2′-O methylation of RNA cap in SARS-CoV-2 captured by serial crystallography
Source: Proc Natl Acad Sci U S A. 2021 May 10;118(21):e2100170118. doi: 10.1073/pnas.2100170118 (PMC8166198; doi:10.1073/pnas.2100170118)
Supplement: Supplementary File [file pnas.2100170118.sapp.pdf]

Supplementary Information for

2′-O Methylation of RNA Cap in SARS-CoV-2 captured by serial crystallography

Mateusz Wilamowski[1,2,3], Darren A. Sherrell[4], George Minasov[5], Youngchang Kim[1,4], Ludmilla Shuvalova[5], Alex Lavens[4], Ryan Chard[6], Natalia Maltseva[1,4], Robert Jedrzejczak[1,4], Monica Rosas-Lemus[5], Nickolaus Saint[6], Ian T. Foster[6], Karolina Michalska[1,4], Karla J. F. Satchell[5], Andrzej Joachimiak[1,2,4*].

[1]Center for Structural Genomics of Infectious Diseases, Consortium for Advanced Science and Engineering, University of Chicago, Chicago, IL, USA [2]Department of Biochemistry and Molecular Biology, University of Chicago, Chicago, IL, USA [3]Department of General Biochemistry, Faculty of Biochemistry, Biophysics and Biotechnology of Jagiellonian University, Krakow, Poland, [4]Structural Biology Center, X-ray Science Division, Argonne National Laboratory, Lemont, IL, USA, [5]Center for Structural Genomics of Infectious Diseases, Northwestern University, Feinberg School of Medicine, Chicago, IL, USA, [6]Data Science and Learning Division, Argonne National Laboratory, Lemont, IL, USA

*Corresponding author – Andrzej Joachimiak

Email: andrzejj@anl.gov

This PDF file includes:

Supplementary Materials and Methods
Figures S1 to S5
Tables S1 to S2
SI References

Materials and Methods

Expression, purification and crystallization of Nsp10/16 complex

The recombinant proteins Nsp10 and Nsp16 from SARS-CoV-2 were expressed in *E. coli* and purified as described by Rosas-Lemus (1). The freshly purified Nsp10 and Nsp16 were mixed in a 1:1 molar ratio to the final concentration of the complex as 2 mg/ml. Subsequently, the Nsp10/16 sample was dialyzed overnight at 295 K in a buffer containing 10 mM Tris-HCl (pH 7.5), 150 mM NaCl, 2 mM $MgCl_2$, 1 mM TCEP, 5% w/v glycerol. The Nsp10/16 heterodimer was supplemented with 2 mM AdoMet (Sigma Aldrich) and concentrated at 21°C to 4.0 mg/ml using a 30 kDa cut-off centrifugal concentrator Amicon Ultra-15 (Millipore). Two criteria for preliminary selection for batch crystallization were getting crystals with the highest possible symmetry, as well as having one Nsp10/16 heterodimer in the asymmetric unit. Such conditions were met by crystals with hexagonal space group $P3_121$. The complex was initially crystallized in a buffer 0.1 M MES (pH 6.5) and 0.6 M sodium fluoride from the Anions Suite crystallization screen (Qiagen). After optimization of crystallization conditions, we used 0.1 M MES pH 6.5, 0.9 M sodium fluoride for batch crystallizations. For each batch of crystals 100 μl of the Nsp10/16 was mixed with 100 μl of the precipitant buffer and crystallization was performed in 500 μl Eppendorf polypropylene tube. These volumes allowed us to get the number of crystals for data collection from one chip. We have prepared several batches using same conditions to get reproducible data collection and have the ability to merge data from multiple chips. The crystals grew to optimum sizes in 10 days at 21°C before the SSX data collection. To obtain the structure of Nsp10/16/Cap-0/AdoMet, the crystal batch was supplemented with 1 mM EDTA two days before data collection.

Preparation of the sample for SSX data collection

The crystals from batch crystallization were centrifuged at 100 RCF for 2 minutes at 295 K. The excess solution was removed with a pipette, until 15 μl was left. Crystals sedimented on the walls of Eppendorf polypropylene tube were gently resuspended using 20 μl pipette tips with a cut end to increase the diameter of the tip and minimize mechanical damage to crystals. Then 1.5 μl of 10 mM stock of the ^{m7}GpppA (S1405L, New England Biolabs) was added to crystal slurry and the mix was loaded on a 60 μm grid made from

nylon (NY6004700, Millipore) which was placed on a 6 μm layer of mylar polyester film. The nylon mesh was covered on the top with a second layer of 6 μm mylar and sealed with the Advanced Lightweight Encapsulator for Serial Crystallography (*ALEX)* magnetic holder, such that the sample is hermetically sealed. Data collection was started approximately 20 minutes after assembly of the chip.

Room-temperature single crystal X-ray crystallography

X-ray diffraction data for the Nsp10/16 in a complex with Cap-0/AdoMet and Cap-1/AdoHcys were collected at 295 K. Crystals were grown by siting drop vapor-diffusion technique. We used CrystalQuick 96-well crystallization plate (Greiner Bio-One) and Mosquito sample dispenser (TTP Labtech) to mix 0.4 µl of crystallization buffer (0.1 M MES (pH 6.5), 0.9 M sodium fluoride) with 0.4 µl of Nsp10/16 (10 mM Tris-HCl (pH 7.5), 150 mM NaCl, 2 mM AdoMet, 2 mM $MgCl_2$, 1 mM TCEP, and 5% w/v glycerol at the concentration of 4.0 mg/ml). Crystals were grown at 295 K for approximately 21 days to reach 250x50x50 µm dimensions. 24 hours prior data collection crystals were soaked with 0.2 mM ^{m7}GpppA (S1405L, NEB) and then were mounted in microRT capillary sleeve (MiTeGen) with a drop of buffer deposited at the end of capillary to prevent a crystal from drying. Single-wavelength x-ray diffraction data were collected at 295 K temperature at the 19ID beamline of the Structural Biology Center at the Advanced Photon Source at Argonne National Laboratory using the program SBCcollect. The diffraction images were recorded on the PILATUS3 X 6M detector at 0.9792 Å wavelength using 0.5° rotation per 0.1 sec exposure at 100% x-ray transmission. The intensities were integrated and scaled with the HKL3000 suite (2). Four data sets were collected and the best data set was selected for structure determination and refinement. Intensities were converted to structure factor amplitudes using truncate (3) program from the CCP4 package (4). The structure was determined by molecular replacement using MolRep (5) software in HKL3000 suite with the Nsp10/16 structure (PDB entry 6XKM) as a search template. The initial solution was refined, using both rigid-body refinement and regular restrained refinement by REFMAC v. 5.8.0258 (6). The best refinement parameters for the Nsp10/16 complex structure were obtained with partial occupancies of 70 and 30 % for Cap-0/AdoMet and Cap-1/AdoHcys, respectively.

Supplementary Figures and Figure Legends

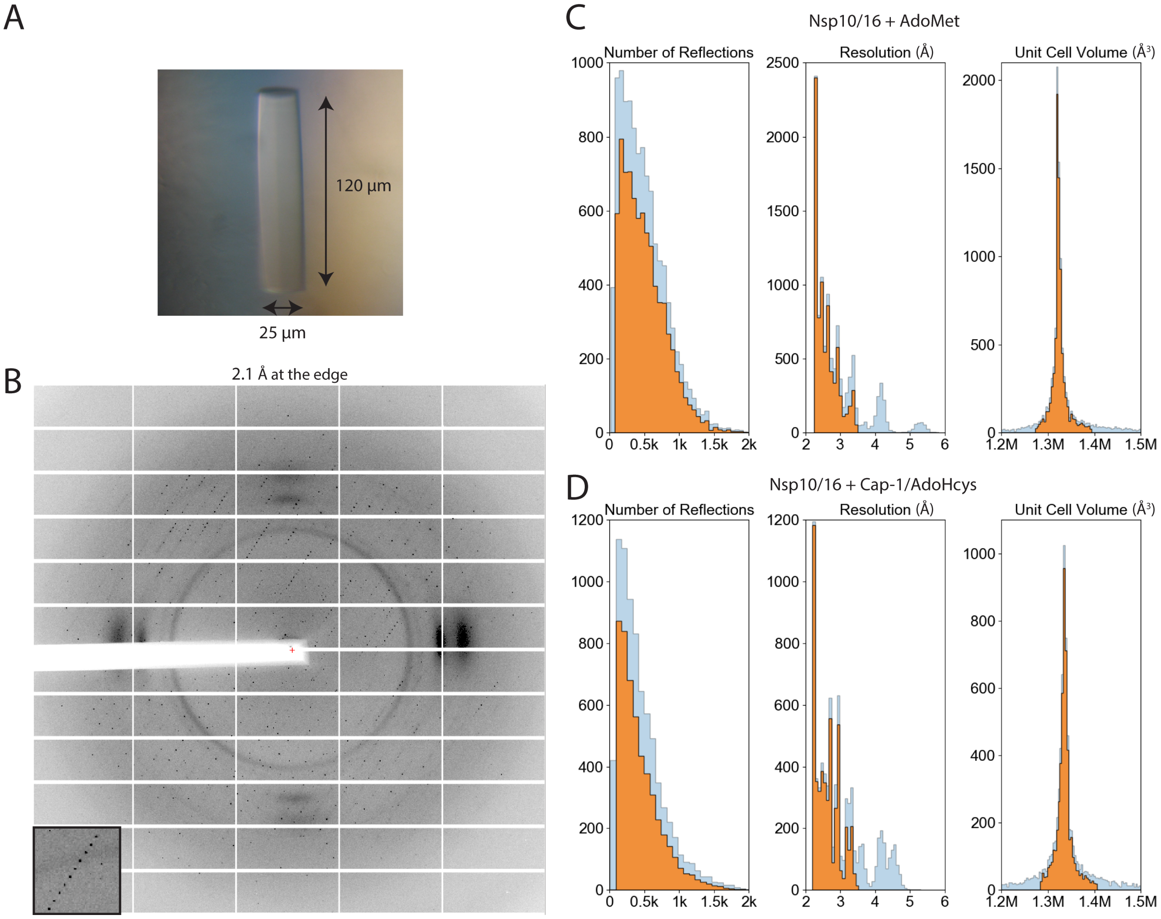

Figure S1. Processing of SSX data for Nsp10/16. (**A**) Representative crystal of Nsp10/16 used for 295 K SSX data collection. (**B**) Diffraction image of one of the Nsp10/16 crystals obtained during SSX data collection, detector corner at 2.1 Å. The fiber diffraction pattern visible at ~ 4 Å originates from the nylon mesh scattering. (**C, D**) Processing of SSX data with DIALS, histograms illustrate number of reflections, resolution and unit cell volume obtained for integrated images. Depicted integration statistics for Nsp10/16/AdoMet (**C**) and Nsp10/16/Cap-1/AdoHcys (**D**). The blue bars show data before rejections of outliers (unit cell deviation > 4.5%, resolution < 3.5 Å, number of reflections < 90), orange bars depict data used for scaling and refinement of the Nsp10/16 structures.

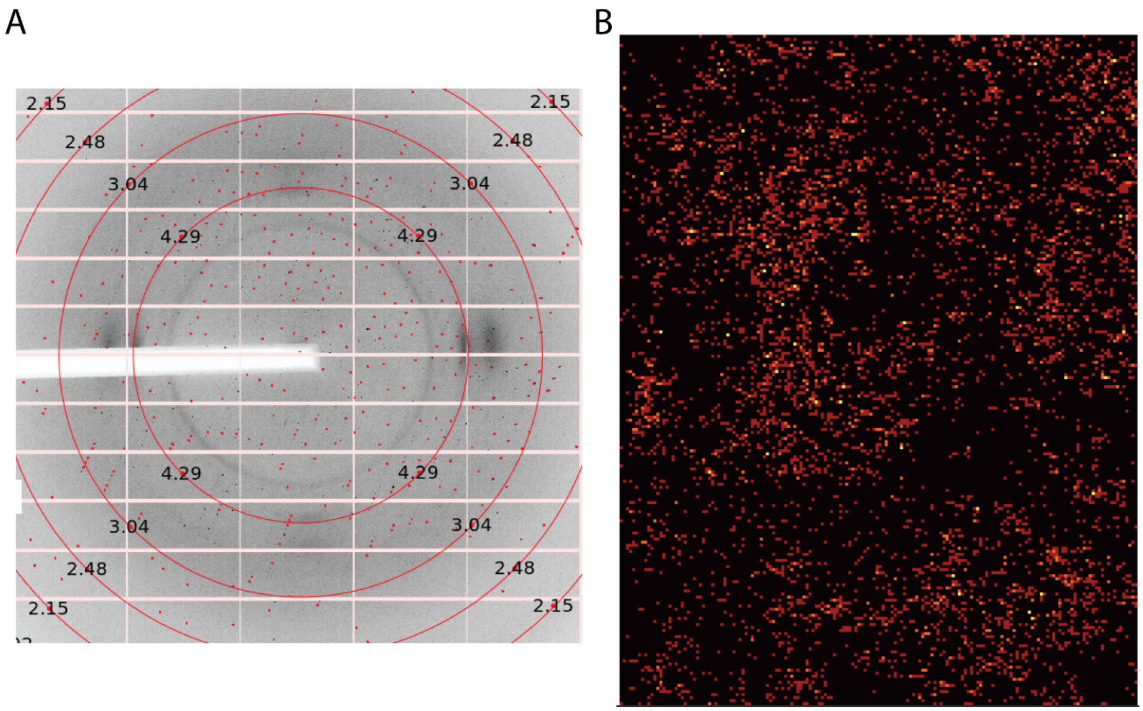

Figure S2. Fixed-target serial crystallography data collection for Nsp10/16 crystals. (**A**) Diffraction image of Nsp10/16 crystals obtained from fixed-target SSX, image processed with DIALS viewer, red dots represent indexed crystal reflections. (**B**) Hit map illustrates images integrated using DIALS, red dots – single lattice, orange dots – two lattices, yellow dots – three lattices determined on integrated crystal diffraction image.

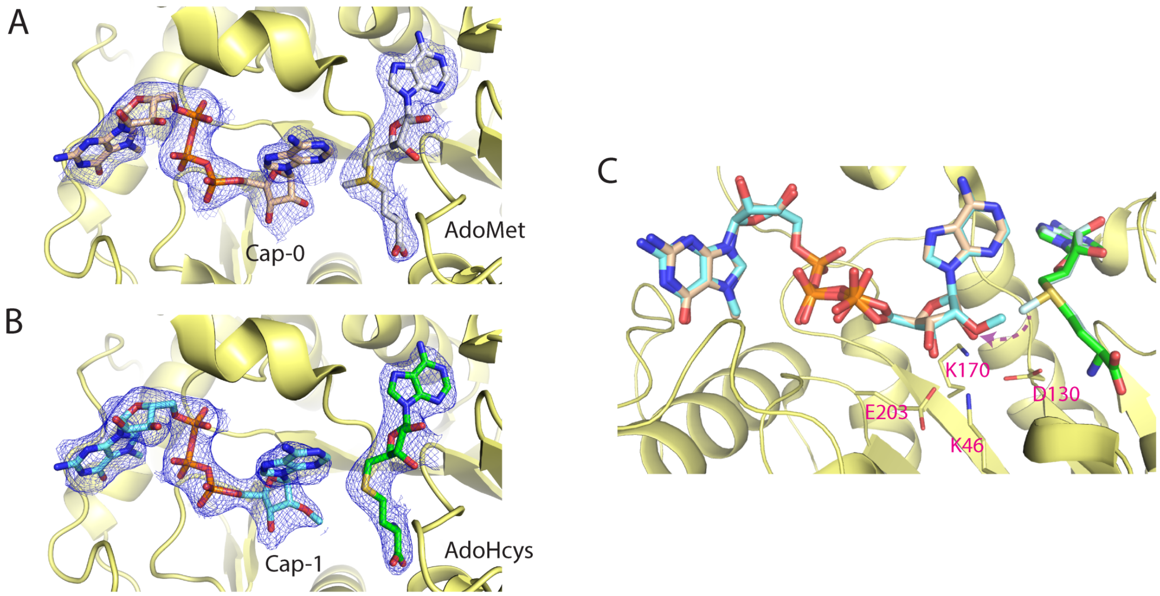

Figure S3. Structures of Nsp10/16 with Cap-0, and Cap-1 at 295K. (**A**) 2mFo-DFc composite omit map for Cap-0 (PDB entry 7JPE). (**B**) 2mFo-DFc composite omit map for Cap-1 (PDB entry 7JHE). Maps are contoured at 1.2 σ, and calculated in CCP4. (**C**) View of the Nsp10/16 active site with mixture of substrates and products observed in single crystal at room-temp (PDB entry 7JIB). Cap-0 (tan) with the 70% occupancy and Cap-1 (aquamarine) with the 30% occupancy.

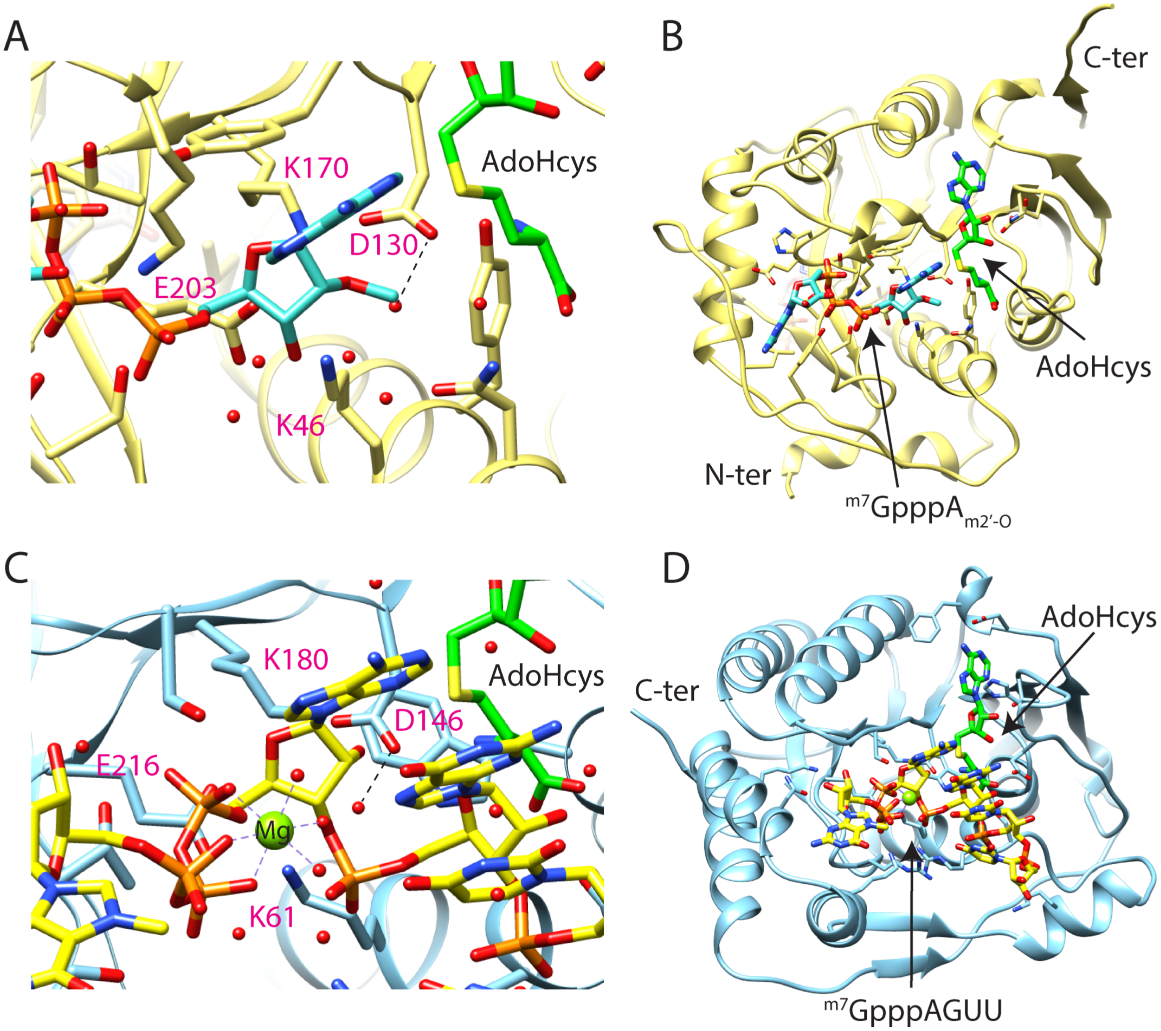

Figure S4. The Nsp16 from SARS-CoV-2 shares structural similarity with the N-terminal fragment of NS5 MTase from Dengue virus. (A) Active site of Nsp16 from SARS-CoV-2 with catalytic tetrad Lys46-Asp130-Lys170-Glu203. (B) Structure of Nsp16 from SARS-CoV-2 in complex with $^{m7}GpppA_{m2'-O}$ (Cap-1) and AdoHcys (PDB entry 7JHE). (C) Active site of the N terminal fragment of NS5 from Dengue virus in complex with ^{m7}GpppAGUU and AdoHcys shares conserved catalytic residues Lys61-Asp146-Lys180-Glu216. (D) The structure of the N terminal fragment of NS5 from Dengue virus (PDB entry 5DTO).

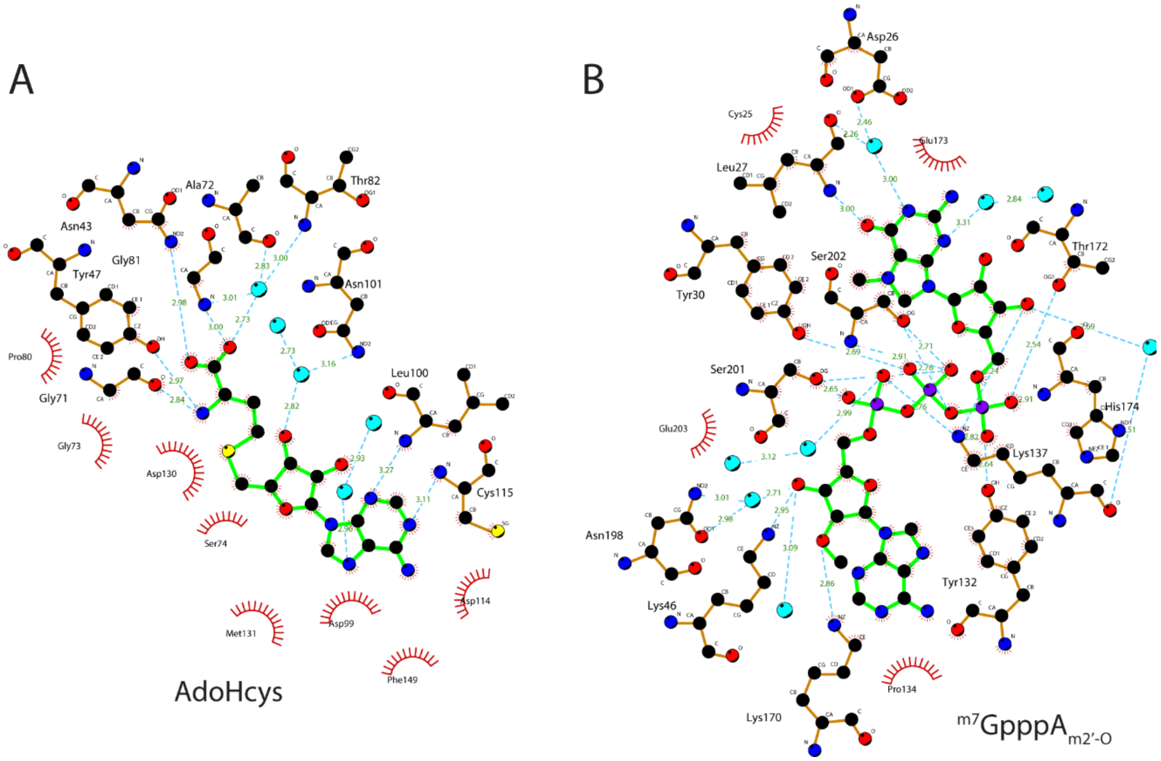

Figure S5. Hydrogen bonds and hydrophobic interactions that stabilize binding of ^{m7}GpppA$_{m2'O}$ and AdoHcys by Nsp10/16 2′-O MTase from SARS-CoV-2. Schematic diagrams of Nsp10/16 interactions with ligands made using LIGPLOT v.4.5.3 software (7).

Supplementary Tables

Table S1. Data collection and processing statistics for Nsp10/16 structures at 295 K.

	Nsp10/16 AdoMet	Nsp10/16 Cap-1 + AdoHcys	Nsp10/16 Cap-0 + AdoMet	Nsp10/16 Cap-0/Cap-1 + AdoMet/AdoHcys
**Data Collection**	fixed-target SSX	fixed-target SSX	fixed-target SSX	single crystal
Crystal holder	60 μm nylon mesh 2 layers of mylar	60 μm nylon mesh 2 layers of mylar	60 μm nylon mesh 2 layers of mylar	MiTeGen microRT capillary
Crystal size (μm)	120x25x25	120x25x25	120x25x25	250x50x50
Diffraction source	APS 19ID	APS 19ID	APS 19ID	APS 19ID
Wavelength (Å)	0.9792	0.9792	0.9792	0.9792
Temperature (K)	295	295	295	295
Beam size (μm)	75 x 75	75 x 75	75 x 75	50 x 50
Exposure time per image (s)	0.05	0.05	0.05	0.2
Beam attenuation	1	1	1	2.2
Crystal – detector distance (mm)	540 (1st chip)<br>319 (2nd chip)	319 (1st and 2nd)<br>446 (3rd chip)	422 (1st and 2nd)	470
Number of chips	2	3	2	-
Number of collected images	73100	105740	74120	180 (90°)
Number of indexed images	10990	7725	5734	180
Indexing hit rate (%)	15.0	7.3	7.8	-
Diffraction images resolution lower than 3.5Å	1622	1654	976	-
Diffraction images < 90 reflections	635	428	187	-
Diffraction images with deviation from the crystal unit cell volume > 4.5%	2009	1831	1362	-
Number of images after rejection of outliers	7951	5038	4704	-
Images with indexed single lattice	6985	4522	4098	180
Images with indexed two lattices	900	488	555	-
Images with indexed three lattices	66	28	51	-
**Lattices merged for structure determination**	7267	4548	4274	180
**Total time of data collection (min)**	180	270	180	< 1
**Dose of X-ray per crystal (MGy)**	0.12	0.12	0.12	4.97

Table S2. Crystallographic data and refinement statistics.

Data Collection	Nsp10/16 AdoMet<br>fixed-target SSX	Nsp10/16 Cap-1 + AdoHcys<br>fixed-target SSX	Nsp10/16 Cap-0 + AdoMet<br>fixed-target SSX	Nsp10/16 Cap-0/Cap-1 + AdoMet/AdoHcys<br>single crystal in capillary
Space group	*P3₁21*	*P3₁21*	*P3₁21*	*P3₁21*
a, *c* (Å)	170.41, 52.63	170.86, 52.80	170.93, 52.79	170.83, 52.74
Resolution range (Å) [a]	49.57 – 2.25 (2.29–2.25)	49.73 – 2.25 (2.29–2.25)	49.72 – 2.18 (2.22–2.18)	50.00 – 2.65 (2.70–2.65)
No. of unique reflections	41,770 (2,090) [a]	42,145 (2,126) [a]	46,273 (2,290) [a]	25,799 (1,263) [a]
Completeness (%)	100.0 (99.7)	100.0 (99.8)	100.0 (99.7)	99.6 (99.8)
Data redundancy	82.41 (10.24)	42.94 (9.28)	50.51 (11.42)	5.2 (4.9)
R_{split} (%) [b]	18.75 (96.45)	26.96 (87.30)	23.44 (98.03)	-
$R_{p.i.m.}$ (%) [c]	-	-	-	7.1 (50.7)
$CC_{1/2}$ [d]	0.968 (0.449)	0.930 (0.473)	0.950 (0.398)	0.981 (0.537)
$I/\sigma I$	2.68 (0.58)	2.45 (0.66)	2.55 (0.53)	15.0 (1.87)
Wilson *B* factor	23.2	21.4	22.8	49.9
**Structure Determination**				
MR initial model PDB ID	6W4H	6WQ3	6WQ3	6WQ3
**Refinement**				
Resolution range (Å)	49.57 – 2.25 (2.31–2.25)	49.73 – 2.25 (2.31–2.25)	49.72 – 2.18 (2.22–2.18)	29.59 – 2.65 (2.72–2.65)
Completeness (%)	99.9 (98.8)	99.9 (99.5)	99.97 (99.74)	99.0 (98.9)
No. of unique reflections	39,686 (2,893)	40,062 (2,962)	46273 (2,290)	24,366 (1,751)
R_{work}/R_{free} (%) [e]	20.65/21.26 (36.3/33.4)	22.31/24.85 (37.4/36.0)	21.70/23.70 (37.9/34.1)	15.49/18.09 (31.2/30.2)
Protein chains/atoms	2/3,204	2/3,210	2/3,210	2/3,244
Ligand/Solvent atoms	30/129	114/160	109/131	189/141
Mean temp. factor ($Å^2$)	37.6	37.1	40.0	55.9
**Coordinate Deviations**				
R.m.s.d. bonds (Å)	0.004	0.005	0.005	0.004
R.m.s.d. angles (°)	1.213	1.363	1.351	1.291
**Ramachandran plot** [f]				
Favored (%)	96.0	95.0	97.0	95.0
Allowed (%)	4.0	5.0	3.0	5.0
Outside allowed (%)	0.0	0.0	0.0	0.0

PDB Accession Code	6XKM	7JHE	7JPE	7JIB

[a]Values in parentheses correspond to the highest resolution shell.
[b]R_{split} as defined by White (8).
[c]$R_{p.i.m}$ as defined by Weiss (9).
[d]$CC_{1/2}$ as defined by Karplus and Diederichs (10).
[e]R = Σh|Fo|–|Fc|/Σh|Fo| for all reflections, where Fo and Fc are observed and calculated structure factors, respectively. R_{free} is calculated analogously for the test reflections, randomly selected and excluded from the refinement.
[f]As defined by Molprobity (11).

Supplementary Information References

1. M. Rosas-Lemus, *et al.*, High-resolution structures of the SARS-CoV-2 2′-O-methyltransferase reveal strategies for structure-based inhibitor design. *Sci. Signal.* **13**, 1202 (2020).
2. W. Minor, M. Cymborowski, Z. Otwinowski, M. Chruszcz, *HKL* -3000: the integration of data reduction and structure solution – from diffraction images to an initial model in minutes. *Acta Crystallogr. Sect. D Biol. Crystallogr.* **62**, 859–866 (2006).
3. J. E. Padilla, T. O. Yeates, A statistic for local intensity differences: Robustness to anisotropy and pseudo-centering and utility for detecting twinning. *Acta Crystallogr. - Sect. D Biol. Crystallogr.* **59**, 1124–1130 (2003).
4. M. D. Winn, *et al.*, Overview of the *CCP* 4 suite and current developments. *Acta Crystallogr. Sect. D Biol. Crystallogr.* **67**, 235–242 (2011).
5. A. A. Lebedev, A. A. Vagin, G. N. Murshudov, Model preparation in MOLREP and examples of model improvement using X-ray data in *Acta Crystallographica Section D: Biological Crystallography*, (International Union of Crystallography, 2007), pp. 33–39.
6. G. N. Murshudov, *et al.*, REFMAC5 for the refinement of macromolecular crystal structures. *Acta Crystallogr. Sect. D Biol. Crystallogr.* **67**, 355–367 (2011).
7. A. C. Wallace, R. A. Laskowski, J. M. Thornton, Ligplot: A program to generate schematic diagrams of protein-ligand interactions. *Protein Eng. Des. Sel.* (1995) **8**, 127 134 (1995) https:/doi.org/10.1093/protein/8.2.127.
8. T. A. White, *et al.*, Crystallographic data processing for free-electron laser sources. *Acta Crystallogr. Sect. D Biol. Crystallogr.* **69**, 1231–1240 (2013).
9. M. S. Weiss, Global indicators of X-ray data quality. *J. Appl. Crystallogr.* (2001) **34**, 130-135 (2001) https:/doi.org/10.1107/S0021889800018227.
10. P. A. Karplus, K. Diederichs, Linking crystallographic model and data quality. *Science (80-.).* **336**, 1030–1033 (2012).
11. I. W. Davis, L. W. Murray, J. S. Richardson, D. C. Richardson, MolProbity: Structure validation and all-atom contact analysis for nucleic acids and their complexes. *Nucleic Acids Res.* **2**, W615-W619 (2004) https:/doi.org/10.1093/nar/gkh398.
